# Employers and the Ex-Service Man

**Published:** 1920-08-21

**Authors:** 


### EMPLOYERS AND THE EX-SERVICE MAN.

Public memory can be bitterly short, but it is hard to believe that within less than two years of the Armistice memory can have played tricks with the definite obligations of the country to ex-Service men. Yet Earl Haig, who has displayed untiring zeal and honest solicitude for the welfare of the men who fought under him, has found it necessary to stir up the sluggish national interest in a subject which ought constantly to be present in the minds of all responsible people until a final solution is reached. In his appeal in *The Times*, Earl Haig, with directness, simplicity, and obvious emotion, asks the nation to provide work for the 200,000 unemployed ex-Service men. Two hundred thousand men—22,000 of them disabled—who risked life and all their material prospects in order to protect this country, want the country to give them just a share in the "fruits of victory." They ask for a chance to work again, and the war has been over nearly two years. At the same time, it must be recognised that a gigantic effort has been required to get back into the social scheme those millions of soldiers who have been demobilised, and its success, so far as it has gone, must not be overlooked in considering the numbers that remain. The last lap is always the stiffest, and it is quite likely that those for whom employment has still to be found are among the more difficult to place. But more can be done by Government, employers, and trade unions, and more must be done.

Let there be no mistake: It is perhaps not inopportune to remind Labour that these organisations have a membership which, in bulk, exceeds that of any other single trade union. Those who treat obligations of honour lightly when the consequences appear to them inconsiderable may therefore be advised to regard the observation of them in this case as—to put it at its lowest—expedient. The Government has worked enthusiastically. Employers in many instances have shown commendably practical insistence upon justice for ex-Service men, and doubtless there are trade unions which have done what they could. But the 19,000 firms on the King's Roll pledged to employ a certain percentage of disabled men are not enough. The 22,000 disabled must be absorbed, and every allowance made for any deficiencies that exist. If Oldham and seven other towns can absorb locally the whole of the disabled in their districts, so can every other town. The employment of the 14,000 ex-officers and other ranks of equivalent educational training ought not to present great difficulty. The remaining 150,000 fit men of the working class constitute a bigger problem, because many of them are unskilled. But it is imperative that those who are willing to work should have the opportunity to work, and here especially the employers and unions must play the game.

The Minister of Labour has made a thoroughly sound proposal that the gaps in the ranks of the building trades should be filled by men from this class, and so help to settle not only that problem, but the housing shortage. But immediately a scheme is put forward, we find the bricklayers and the carpenters and joiners strongly opposing dilution, and it is notorious that many similar obstacles have been set up in other trades. Is it too late or too hopeless to appeal to the trade unions to practise a little of the idealism they so constantly profess, by making some reasonable effort to concede the right to work to men of their own class without whose campaigning they would have lost every trade union principle under which they carry on to-day? The case of the building trades, which must be occupied for years in making up the leeway of five years of war, is particularly flagrant, and hard to speak of with moderation or patience.

We hope the hospitals throughout the country will bear the subject in mind upon every occasion. They do not employ large numbers of males, but there are many places which can be filled by unskilled though handy and reliable men, and there are many posts which could quite well be undertaken by disabled men. If there are those who consider it impossible, let them at least determine to employ a fair percentage of fit ex-Service men, so long as any remain workless. It is not conceivable that sympathy for them can be lacking in hospitals, for there the courage, spirit, and sacrifice of the soldier under suffering is a matter of knowledge and experience.

